# From Endosymbiont to Host-Controlled Organelle: The Hijacking of Mitochondrial Protein Synthesis and Metabolism 

**DOI:** 10.1371/journal.pcbi.0030219

**Published:** 2007-11-02

**Authors:** Toni Gabaldón, Martijn A Huynen

**Affiliations:** Nijmegen Center for Molecular Life Sciences, Center for Molecular and Biomolecular Informatics, University of Nijmegen, Nijmegen, The Netherlands; University of California San Diego, United States of America

## Abstract

Mitochondria are eukaryotic organelles that originated from the endosymbiosis of an alpha-proteobacterium. To gain insight into the evolution of the mitochondrial proteome as it proceeded through the transition from a free-living cell to a specialized organelle, we compared a reconstructed ancestral proteome of the mitochondrion with the proteomes of alpha-proteobacteria as well as with the mitochondrial proteomes in yeast and man. Overall, there has been a large turnover of the mitochondrial proteome during the evolution of mitochondria. Early in the evolution of the mitochondrion, proteins involved in cell envelope synthesis have virtually disappeared, whereas proteins involved in replication, transcription, cell division, transport, regulation, and signal transduction have been replaced by eukaryotic proteins. More than half of what remains from the mitochondrial ancestor in modern mitochondria corresponds to translation, including post-translational modifications, and to metabolic pathways that are directly, or indirectly, involved in energy conversion. Altogether, the results indicate that the eukaryotic host has hijacked the proto-mitochondrion, taking control of its protein synthesis and metabolism.

## Author Summary

Mitochondria are compartments from the eukaryotic cell that originated from the endosymbiosys of an alpha-proteobacterium. The bacterial-like metabolism of this early endosymbiont was thought to differ substantially from that of modern mitochondria, but so far we do not know the details of this bacterium-to-organelle transformation. To address this issue, we used an evolutionary approach to find genes derived from the ancestor of mitochondria. By identifying eukaryotic genes that are closely related to alpha-proteobacterial ones, we reconstructed a set of genes derived from the mitochondrial ancestor. We used that set to infer the ancestral mitochondrial metabolism, and subsequently compared it with those of modern mitochondria, as reconstructed from proteomics data from yeast and human. This allowed us to trace the metabolic evolution of mitochondria. What we found is that there has been a large turnover of the protein content of mitochondria, which has affected some pathways more than others. Pathways for protein synthesis and those involved in energy conversion have been preferentially retained in the mitochondrion, whereas those involved in replication, transcription, cell division, transport, regulation, and signal transduction have been replaced by eukaryotic proteins. Our findings show how the eukaryotic host has taken control of the endosymbiont, effectively hijacking those pathways that it could use.

## Introduction

Mitochondria are organelles that are found in virtually all eukaryotic cells. In addition to their role in energy conversion, mitochondria are involved in many processes from intermediate metabolism, such as synthesis of heme groups [1], steroids [2], amino acids, and iron-sulphur (Fe-S) clusters [3]. Phylogenetic analyses of mitochondrial genes indicate that all mitochondria derive from a single alpha-proteobacterial ancestor, the so-called proto-mitochondrion [4]. During the transformation of proto-mitochondrion to organelle, its proteome underwent a series of modifications, including, among others, the acquisition of a protein import machinery and an ADP/ATP carrier, leading to a situation in which only a minority of mitochondrial proteins can be traced back to an alpha-proteobacterial ancestor [5,6]. Similarly, large transformations of the mitochondrial metabolism are thought to have occurred in the course of mitochondrial evolution [7,8]. According to a recent reconstruction [9], the proto-mitochondrion possessed an aerobic metabolism comprising a considerable variety of pathways, such as fatty-acid synthesis and degradation, the respiratory chain, and the Fe-S cluster assembly pathways. Some studies have focused on the subsequent evolution from the alpha-proteobacteria of some mitochondrial pathways such as the electron transport chain [10,11]. However, no comprehensive analysis has been performed so far to analyze the proteomic transition of mitochondria at a larger scale. It is still largely unknown, for example, which aspects of the proteome of modern mitochondria resemble that of its bacterial ancestor or to what extent the current metabolic diversity observed in mitochondria from different organisms was achieved through the differential gain or differential loss of proteins.

To address these questions, we compared ancient and modern mitochondrial proteomes and their inferred metabolic pathways. To reconstruct the proteome of the proto-mitochondrion, we have used a similar approach to the one used previously for a smaller set of genomes [9]. The rationale behind this approach is that proto-mitochondrial proteins are eukaryotic proteins with an alpha-proteobacterial ancestry and that they can be detected by constructing phylogenies of eukaryotic proteins and examining those for a monophyletic relation between alpha-proteobacterial proteins and eukaryotic proteins. Metabolic pathways from modern mitochondria were inferred from recent proteomics surveys of highly pure, isolated mitochondria from yeast and human. A comparison of the functional classification of these proteomes indicates that only in classes corresponding to translation, post-translation modification, and protein folding and metabolism do current-day mitochondria resemble the proto-mitochondrion. Other classes have either disappeared or have been replaced by proteins of non(detectable) alpha-proteobacterial origin.

Focusing on the metabolic transition, we compared the inferred ancestral mitochondrial metabolism with the metabolism of present-day mitochondria as it can be inferred from comprehensive mitochondrial proteomics. By comparing the three reconstructed metabolic pathways, we trace the main lines of the metabolic transition from the early endosymbiont to the modern organelle, as well as the later divergence of fungal and metazoan mitochondrial metabolic pathways. Altogether, our results indicate a continuously increasing bias toward energy conversion from the alpha-proteobacteria to the proto-mitochondrion, and from the proto-mitochondrion to current-day mitochondrion, a significant retargeting of metabolic enzymes of alpha-proteobacterial origin to other cellular compartments and a complete eukaryotic takeover of replication, transcription, mitochondrial division and signal transduction, and gene regulation.

## Results/Discussion

### Reconstruction of the Proto-Mitochondrial Proteome

To reconstruct the ancestral proto-mitochondrial proteome, we performed a phylogenomics analysis of 11 alpha-proteobacterial genomes, among a total of 144 complete genomes, including those of 16 eukaryotes. Compared to our previous study [9] that included 77 genomes of which nine were eukaryotes and six alpha-proteobacteria, this represents a significant increase in the amount of data to be analyzed. The analysis involved the retrieval of protein families with alpha-proteobacterial and eukaryotic members, and the reconstruction of their phylogenetic trees to scan for those indicating a monophyletic origin of eukaryotic and alpha-proteobacterial proteins (see Materials and Methods). First, the phylomes of the 11 alpha-proteobacteria were derived using neighbor joining (NJ). For those protein families whose NJ-tree topology supported a proto-mitochondrial origin (1,026 families, NJ-set), maximum likelihood (ML) trees were derived using PhyML and scanned, producing a subset of 842 families (ML-set) whose proto-mitochondrial origin is supported by both tree-reconstruction methods. We consider these to be minimal estimates of the ancestral proto-mitochondrial proteome because: (1) genes may have diverged too far to be reliably identified by homology or phylogeny analyses, and (2) our procedure cannot recover genes that have been lost from either all the alpha-proteobacterial genomes considered or all the eukaryotic genomes considered, like the bacterial RNA polymerase that thus far has only been found in the mitochondrial genome of Reclinomonas americana [12] and FtsZ, which has only been retained in protists [13].

To roughly estimate the accuracy and sensitivity of our method, we benchmarked our procedure by using the mitochondrial genome of R. americana and the genome of the bacterium Deinococcus radiodurans. The jakobid R. americana possesses the mitochondrial genomes with the highest number of genes [12], encoding 67 proteins that presumably have an alpha-proteobacterial origin and thus can be used as a “gold standard” to test the sensitivity of our method (Table 1).

**Table 1:**
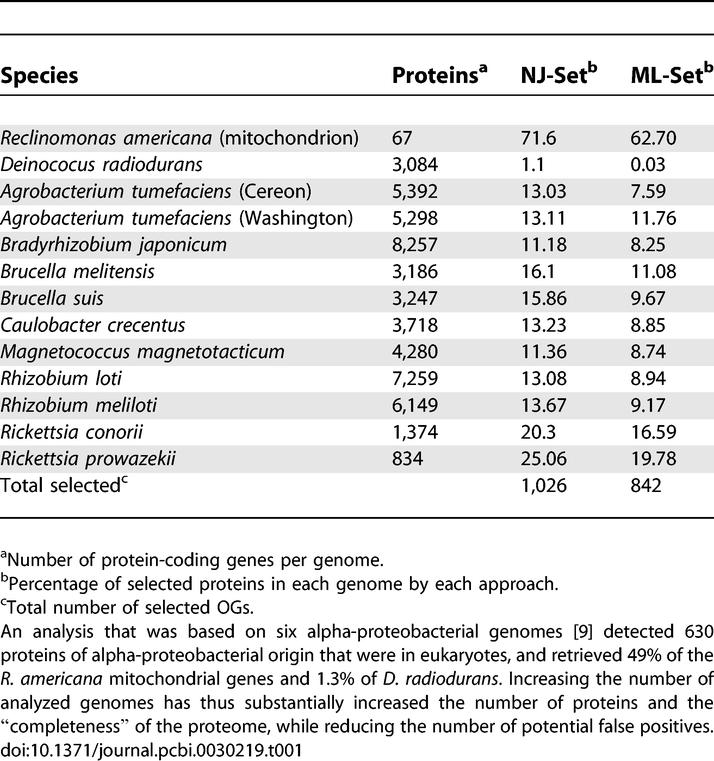
Selected OGs and Benchmarking of the Different Approaches

Our procedure retrieved the majority of R. americana mitochondrial-encoded proteins 71.6% (NJ-set) and 62.7% (ML-set). In addition, to estimate the fraction of false positives, we used the bacterium D. radiodurans, which has no direct relation to the eukaryotes [14]. Here, out of a total of 3,085 proteins, our procedure selected only 34 (1.1%) in the NJ-set and 1 (0.03%) in the ML-set. Taken together, these results indicate that both sets have a high accuracy and a reasonable sensitivity. Compared to an earlier estimate [9], we observe a substantial improvement in terms of coverage and potential false positives (Table 1) due to a doubling of the number of genomes compared. Despite this increase in terms of sensitivity and coverage, the overall picture of the proto-mitochondrial metabolism remains similar to that which has previously been reported [9]. Nevertheless, the increase of coverage and sensitivity has had a positive effect on the completeness of the pathways recovered, which can now be studied in more detail (see discussions below). Besides reconstructing the proto-mitochondrial metabolism with higher resolution, we have focused here on the metabolic changes that occurred to the proto-mitochondrion during the process of transformation into a modern organelle.

### Comparative Analysis of Present and Past Mitochondrial Proteomes

In order to compare the overall functional diversity of the reconstructed metabolic pathways, and therefore trace the metabolic transition of mitochondria, we used the Clusters of Orthologous Groups (COG) database functional classification scheme [15,16] to classify the considered proteomes (Figure 1). In the proto-mitochondrion, the largest fractions of proteins with known function are devoted to energy conversion (13.8%), amino acid metabolism (14.3%), and protein synthesis (9.6%). Compared to the free-living alpha-proteobacteria Caulobacter crescentus (6%, 8.5%, and 10%, respectively) and Mesorhizobium loti (7.2%, 16%, and 4.4%) or the parasitic species Rickettsia prowazekii (11.7%, 4.3%, and 20.2%), the major bias in the proto-mitochondrion is toward energy conversion and the metabolism of amino acids. Conversely, processes such as cell division (1%) or signal transduction (1%) are nearly nonexistent, and have very likely been extensively lost from the eukaryotes or the alpha-proteobacteria considered after the endosymbiosis event. A similar functional bias toward energy conversion, amino acid metabolism, and protein synthesis is also found in modern mitochondria. However, here the bias appears stronger, specifically toward energy conversion and toward protein synthesis and folding that together represent more than the 50% of the proteins with known function (as compared to 28% in the proto-mitochondrion). That the functional bias of present-day mitochondria is more pronounced than that of the proto-mitochondrion is confirmed by calculating the entropy (*H*) of the distribution of proteins among functional classes (*H* = −Σ(*P_i_* × log *P_i_*), where *P_i_* is the relative frequency of the class *i*). The entropy is lower (the distribution is more dominated by a few frequencies) in yeast (*H* = 0.79) and human (*H* = 0.95) than in the proto-mitochondrion (*H* = 1.09), confirming an increase in the level of specialization, which is most pronounced in yeast. As part of this specialization, the functional classes of amino acid metabolism and secondary metabolism have been significantly diminished, whereas “carbohydrate metabolism and transport” or “cell envelope biogenesis” have virtually disappeared.

**Figure 1:**
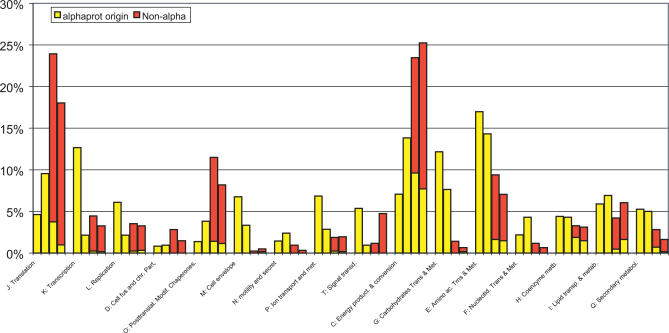
Relative Weights of the Functional Classes in the Proteomes From left to right, Mesorhizobium loti, the proto-mitochondrion, yeast mitochondria, and human mitochondria. The colors in the bars indicate the origin of the proteins in that functional class for that given organism (yellow: alpha-proteobacterial origin. red: other origin). We used the NJ-set as a reference to calculate the fraction that is evolutionary-derived from the proto-mitochondrion. Functional classes are derived from COG [16]. Alpha-proteobacterial–derived proteins are a minority in all classes except Coenzyme biosynthesis; the energy production/conversion class is the most “alpha-proteobacterial–derived.”

### A Starting Point: A Diverse Proto-Mitochondrial Metabolism

Intrigued by the dominance of metabolism in the proto-mitochondrion, we mapped the annotated functions of the selected orthologous groups onto the metabolic maps of the KEGG: Kyoto Encyclopedia of Genes and Genomes pathways database [17], and reconstructed the proto-mitochondrial metabolism (Figure 2). Pathways that are shown in Figure 2 have several consecutive steps present in the most stringent ML-set and have been completed or extended with adjacent reactions from the NJ-set. The notable presence of enzymes from oxidative phosphorylation (28 orthologous groups [OGs]) and beta-oxidation (seven OGs) clearly indicate that the proto-mitochondrion had an aerobic metabolism in which the latter could have provided the former with NADH and FADH_2_. These two pathways together with lipid synthesis, biotin, vitamin B6, heme synthesis, and Fe-S cluster assembly can be reconstructed almost completely. Do note that biotin and vitamin B6 are required for heme synthesis. Except for lipid synthesis, the most complete metabolic pathways can therefore be linked either directly or indirectly to oxidative phosphorylation. In contrast, some mitochondrial pathways, such as the citric acid cycle, appear incomplete, whereas the urea cycle is absent. Previous work on the origin of the citric acid cycle in yeast [18,19] shows a complex phylogeny for this group of proteins, which is consistent with our results. Notably, the part of the incomplete citric acid cycle predicted by our analyses also exists in present-day organisms such as *Chlamydia* [19,20], and can be used for the catabolism of glutamate via 2-oxoglutarate. However, based on these results, we cannot exclude that the citric acid cycle was complete in the proto-mitochondrion, but that later in its evolution, some of its enzymes have been replaced by proteins of a different, non–alpha-proteobacterial origin.

**Figure 2:**
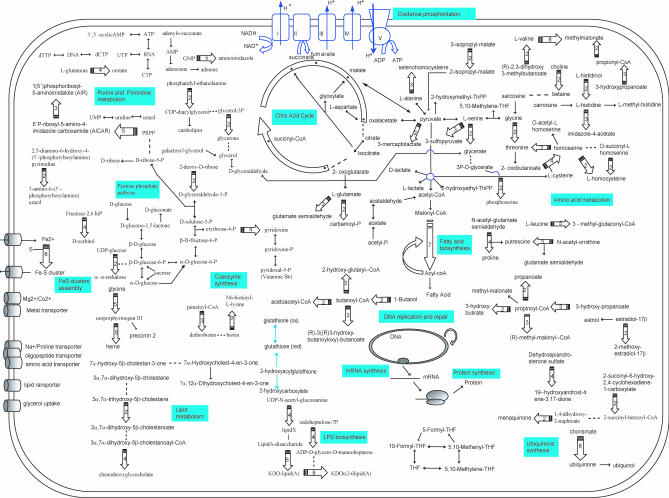
An Overview of Metabolism and Transport in the Proto-Mitochondrion Metabolism and transport were deduced from the OGs present in the estimated proteome (see text). Boxes, arrows, and cylinders indicate pathways, enzymes, and transporters, respectively. Several consecutive steps can be condensed into a bigger arrow with a number indicating the steps included. Single missing steps connecting recovered pathways are indicated as dashed lines.

As expected [9,21], the glycolytic pathway is not of alpha-proteobacterial descent, but we do find some steps from fructose and mannose metabolism, such as fructose-2,6-biphosphatase and mannose-6-P isomerase, and a considerable number of connected steps from the pentose phosphate pathway, such as transketolase and deoxyribokinase (Figure 3). Pathways from pentose phosphate metabolism could have provided the proto-mitochondrion with intermediates for the anabolism of amino acids, vitamins, and nucleotides. Indeed, the synthesis of erithrose-4-P from glucose provides the link between the reconstructed pentose phosphate pathway and vitamin B6 synthesis (Figure 2). Nucleotide metabolism (23 OGs) is also well-represented in the proto-mitochondrion, but in contrast to the above-mentioned pathways, contains mainly “isolated enzymes” (for the exceptions, see Figure 3), and its pathways are far from complete. From amino acid metabolism (60 OGs), we recover many stretches of interconnected steps, separated by some gaps. Some of the amino acid metabolism enzymes in the proto-mitochondrion, such as threonine synthase, threonine dehydratase, and L-serine dehydratase, are specifically involved in the interconversion of amino acids, indicating a potential to convert certain amino acids to others. Furthermore, the above-mentioned vitamin B6 is needed as a cofactor by enzymes that catalyze transaminations and other reactions of the amino acid metabolism, indicating a certain level of consistency in the reconstructed metabolism.

**Figure 3:**
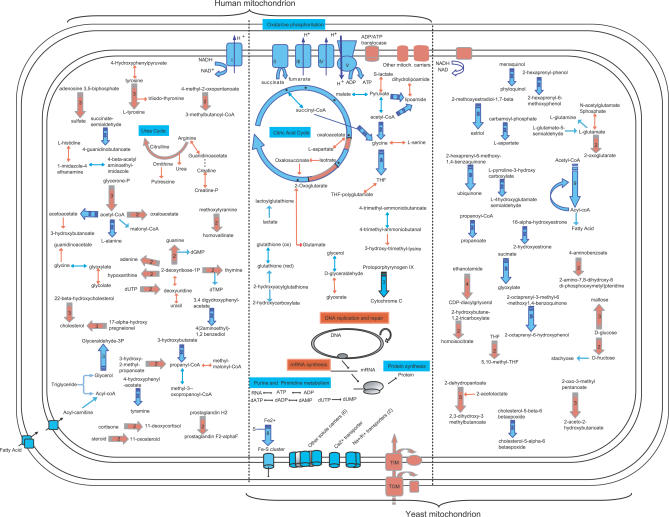
Reconstructed Human and Yeast Mitochondrial Metabolic Pathways Human (left) and yeast (right) metabolic pathways were deduced from the function of the proteins compiled in the MitoProteome Dataset [25] and present in the yeast proteomics set [24], respectively. In order to facilitate the comparison of both metabolic pathways, pathways shared by the two species are depicted in the middle region of the figure, pathways at the extremes of the dashed lines are exclusive for human (left) or yeast (right) mitochondria. Color codes indicate whether the pathway was likely present in the proto-mitochondrion (blue) or has a different origin (red). Only those pathways with two or more consecutive steps are depicted. Symbols are as in Figure 2. All proteins are nuclear-encoded except for nad1–6 subunits of Complex I in human; the atp9 subunit of Complex V in yeast; and the Cob subunit in Complex III, cox1–3 subunits of Complex IV, and the atp6 and atp8 subunits from Complex V in both species.

The abundance of metabolite transporters suggests a host dependency of the proto-mitochondrion. Of the cation transporters, the Fe^2+^ importer is particularly interesting because it could have provided the iron for the Fe-S cluster assembly pathway. Also, the protein that is required for Fe-S clusters in the cytoplasm (ATM1) appears to have been present in the proto-mitochondrion. There are several other cation transporters (Mg^2+^/Co^2+^ and K^+^) that could have been used either to maintain the ion homeostasis or to obtain the cofactors needed for the enzyme activities. The emerging picture thus is that of a (facultatively) aerobic endosymbiont catabolizing lipids, glycerol, and amino acids provided by the eukaryotic host. From the host point of view, although energy conversion has been a dominant factor throughout the evolution of the mitochondria, this appears not to have been the sole benefit from the early symbiotic relationship.

### Two Versions of a Modern Mitochondrial Metabolism: Yeast and Human

How similar are modern mitochondria to their common ancestor? To address this question, we need to compare the ancestral proteome to its modern counterparts. Although it might appear that it is easier to obtain information from modern mitochondria than from the extinct proto-mitochondrion, the fact is that this has been possible only recently. Progress in subcellular proteomics techniques has provided the means to approximate the full identification of the protein complement of mitochondria [22,23]. As a consequence, large datasets of mitochondrial proteomes of several model organisms have been published in recent years [24–28]. For their relevance and high coverage, we have chosen the MitoProteome database of experimentally identified human mitochondrial proteins [25] and a mass spectrometry analysis of highly pure isolated yeast mitochondria [24], including 847 and 743 proteins, respectively. We used the annotated functions of the proteins in these sets to infer (see Materials and Methods) the metabolic pathways of present-day mitochondria from yeast and human (Figure 1). Both reconstructed metabolic pathways, and especially that of human mitochondria, should be regarded as incomplete, because the proteomics sets do not present full coverage and some of the identified mitochondrial proteins do not have a (predicted) function. It must be noted, therefore, that some of the observations may be subject to change as new annotation and localization data become available. Nevertheless, we consider both sets to be highly informative and representative enough to provide an overall picture of the metabolic composition of modern mitochondria.

Remarkably, the overlap between the human and yeast mitochondrial proteomes is rather modest with 312 (42%) of the yeast mitochondrial proteins having 400 (47%) orthologs in human mitochondria. This difference can be real, indicating metabolic differences between human and yeast mitochondria, but can also be explained by an incomplete coverage of both sets. At least for the observed large fraction of human mitochondrial proteins that cannot be found in yeast mitochondria, we expect the lack of coverage to have a minor effect, because the yeast proteome set used here is estimated to account for about 80%–90% of the total proteome [5,24]. In addition, in many cases, the lack of yeast mitochondrial orthologs of human mitochondrial proteins affects complete pathways, or there is no ortholog in the complete yeast genome. Of the 447 human mitochondrial proteins that do not have a yeast mitochondrial counterpart, 377 (79%) do not have an ortholog in the yeast genome. This indicates that, besides a common core of functions, there is significant metabolic differentiation in the mitochondria of these two species. Some of these differences and similarities between human and yeast mitochondria are revealed by an automatic analysis of biological process gene oncology (GO) terms significantly enriched or specific in the different proteomes fractions (Figure 4). For instance, processes such as Induction of apoptosis or C21-steroid hormone metabolism appear specific to the fraction of the human proteome that is not shared with yeast mitochondrion and that has no alpha-proteobacterial origin. Conversely, processes specific to yeast mitochondrion include Energy reserve metabolism and Monosaccharide transport, whereas Isoprenoid biosynthesis or Carboxylic acid metabolism, among others, are shared by yeast and human mitochondrion in their non–protomitochondrial-derived fraction. Finally, processes that are significantly enriched in the common core of both proteomes that is conserved from the proto-mitochondrial ancestor are Generation of precursor metabolites and Energy and Cofactor metabolism.

**Figure 4:**
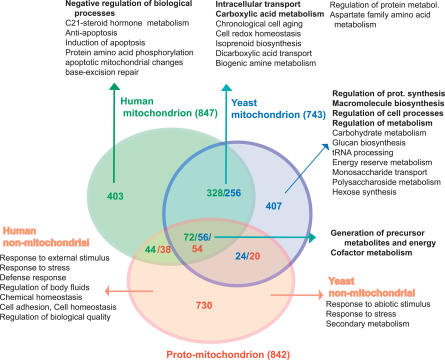
Venn Diagram Representing the Overlap of the Three Considered Proteomes The human mitochondrial proteome (green) [25], the yeast mitochondrial proteome (blue) [24], and the reconstructed proto-mitochondrial proteome (brown). For each proteome, the number of proteins in each fraction is indicated. The numbers of proteins in a single fraction vary because there are varying numbers of (in-)paralogs between the species within the same OG. Arrows from each fraction point to lists of biological process GO terms that are significantly enriched (bold) or specific to that fraction (see Materials and Methods). No significantly over-represented terms were found in the proto-mitochondrial–derived fractions of the mitochondrial proteome, likely due to the fact that most of their pathways (e.g., electron transport chain) also have components of eukaryotic origin.

The observed differences between yeast and human proteomes are the result of a combination of differential gain and loss processes. Differences in the fractions derived from the alpha-proteobacteria are clearly the result of differential loss (e.g., Complex I) or retargeting (e.g., fatty acid oxidation) of proteins. In contrast, to assess whether the differences in the rest of the proteome are mainly due to differential gain or loss would require the mitochondrial proteomes from a wider variety of species to be able to reconstruct intermediate ancestral states.

By having a more detailed look at the specific metabolic activities, examples of specific pathways and complexes can be found (Figure 3). For instance, examples of activities that are present in human mitochondria but absent from their yeast counterparts are NADH:ubiquinone oxidoreductase (Complex I) [11], fatty acid beta-oxidation [29], steroid biogenesis [2], and the apoptotic Bcl2-family signaling pathway [30]. Conversely, glycerone-P metabolism, trehalose synthesis, and starch and sucrose degradation appear to be specific for yeast mitochondria. Many other pathways, such as most of the oxidative phosphorylation, Fe-S cluster assembly, most of the proteins of the mitochondrial carrier family, and the protein synthesis and import machineries, are shared by mitochondria from both organisms.

### A Major Proteome Turnover during Mitochondrial Evolution

The overall similarity between the proto-mitochondrion and modern mitochondria in the functional classification of their proteins is particularly striking when one realizes that there has been a massive turnover of proteins. Indeed, previous analyses of modern mitochondrial proteomes [9,31] have shown that only a minor fraction of them have a clear alpha-proteobacterial ancestry. This extensive turnover is confirmed in our present analysis, although the use here of broader proteomics sets and different phylogenetic approaches introduces variations in the estimates. Quantitatively, only 16.3% (138 proteins) and 12.6% (94) of the human and yeast mitochondrial proteomes, respectively, can be traced back to the protomitochondrion if we use the NJ-set as a reference. When the more stringent ML-set is used, these percentages are reduced to 13.7% (116) and 10.8% (80), respectively. These percentages are fairly similar to those found in our previous study for yeast (16%) and human (14%). The low fraction of proto-mitochondrial proteins in modern mitochondria is the result of the combination of a proteome reduction and a proteome expansion process [5,32]. Firstly, some proto-mitochondrial pathways, such as LPS-biosynthesis or lipid synthesis, have been lost from the mitochondrion and moved to other parts of the cell. Secondly, new proteins have been recruited to the mitochondrion by the gain of novel pathways, such as the protein import machinery [33] or the mitochondrial carrier family that includes the ADP/ATP carrier. These two processes are accompanied by a parallel expansion and reduction of the corresponding metabolic capacities. In addition, the amelioration of some pathways, such as the recruitment of new subunits to Complex I [11] and other electron transport chain complexes [10], would have contributed to the proteome renewal without significantly altering the metabolic capacities of the organelle. Although it can be considered an ongoing process, the proteome turnover of mitochondria is likely to have been very extensive in the early stages of eukaryotic evolution. This is illustrated by the fact that even in the common core of the human and yeast mitochondrial proteome, only 18% of the proteins are of alpha-proteobacterial descent. Most of the common pathways do have a proto-mitochondrial origin, but an extensive incorporation of new subunits before the divergence of the human and yeast lineages results in a significant amount of non–alpha-proteobacterial components in these pathways. For instance, about half of the proteins in the electron transport complexes shared by yeast and human have non–alpha-proteobacterial origin. The recruitment, before the radiation of opisthokonts, of new pathways of non–alpha-proteobacterial origin, such as the protein import and mitochondrial division machineries or the ADP/ATP transport system, together with the differential loss of proto-mitochondrial pathways in the fungal and metazoan lineages, would have also contributed to the enrichment in non–alpha-proteobacterial proteins of the mitochondrial core.

Our results (Figure 1) indicate that the proteome turnover has affected some functional classes more than others. For instance, the fraction of alpha-proteobacterial–derived proteins is larger in classes such as coenzyme metabolism (57% in yeast and 47% in human) or energy production and conversion (41% and 30.6%) than in classes such as translation (15.7% and 5.4%) or protein turnover and chaperones (12% and 14%).

The (almost) complete renewal of classes such as cell division and fusion, transcription, replication, and signal transduction is consistent with the fact that a major difference of the early endosymbiont and present-day mitochondria is that the latter have lost their autonomy, having come under the full control of the host. Although chloroplasts usually have retained a bacterial-type division machinery, most mitochondria use a completely eukaryotic-derived system [34], something that could have facilitated the control of the number and shape of mitochondria in a cell.

Besides the major role that the above-mentioned proteome turnover has played in the transition of mitochondria, there are other mechanisms that might have contributed to this process. One such mechanism that has apparently been important in mitochondrial evolution is the recruitment to new functions of some proteins already present in the endosymbiont. Such is the case for the protein import machinery, of which some components, including most of the soluble chaperones that assist in the process, have homologs in bacteria [35,36]. Another case of gain of function of ancient proteins is illustrated by six of the so-called supernumerary subunits of the NADH:ubiquinone oxidoreductase (Complex I), whose origin can be traced back to the alpha-proteobacteria, but whose association with the complex is restricted to the eukaryotes [11].

Another result that is consistent with the view of a metabolic hijacking of the proto-mitochondrion is the significant fraction of proto-mitochondrial proteins that have been retargeted to other organelles in the course of eukaryotic evolution, confirming earlier results [9]. In the present set, non-mitochondrial proteins represent more than 50% (68%, 246 proteins, in human; and 57%, 106 proteins, in yeast) of the total set of alpha-proteobacterial–derived proteins in the cell. As in the case of the mitochondrial proteome turnover, the process of retargeting has also affected some classes more than others. For instance, from the 41 yeast proto-mitochondrial–derived proteins in our NJ-set whose mutants specifically impair respiration according to a large-scale analysis in yeast [37], 36 (88%) have a mitochondrial localization. This indicates that most of the respiratory metabolism donated by the mitochondrial ancestor has remained inside the organelle. In contrast, larger fractions of carbohydrate and nucleotide metabolic pathways that can be traced back to the proto-mitochondrion have been retargeted during evolution. This fraction includes complete pathways or part of them, such as the initial steps from the synthesis of uridine monophosphate (UMP) [38], which is cytosolic in human and yeast; biotin synthesis and fatty-acid beta-oxidation in yeast, which are cytosolic and peroxisomal, respectively, in human; and lipid synthesis, which is cytosolic in human.

### Concluding Remarks

The evolutionary analysis of the mitochondrial proteome reveals a continuous functional shift toward specialization in energy metabolism that already started in the endosymbiotic phase. This specialization has been achieved despite a major turnover of the proteome that has reduced the alpha-proteobacterial fraction of the mitochondrial proteome to a modest 10%–16%. This proto-mitochondrial fraction is nearly completely devoid of functional classes such as signal transduction and classes involved in mitochondrial fission and fusion, suggesting that the alpha-proteobacterial proteins performing such functions were early substituted by eukaryotic proteins, providing the eukaryotic host with effective control of the mitochondria. The extent of this hijacking of the proto-mitochondrial metabolism is such that a large fraction of metabolic enzymes have been retargeted to other compartments of the cell. Altogether, the results indicate that most of what remains of the proto-mitochondrion is a bacterial-derived metabolism that is under the full control of the eukaryotic proteome. In the course of mitochondrial evolution, the metabolism got more biased toward energy metabolism and protein synthesis, diminishing functional classes, such as amino acid and nucleotide metabolism. The processes of protein gain, loss, and retargeting have acted in a lineage-specific manner, resulting in the metabolic differences encountered between human and yeast mitochondria. Pathways that are common to most mitochondria and are not of proto-mitochondrial origin, such as the protein import machinery and the mitochondrial carrier family, have likely played a key role in the bacterium-to-organelle transition of mitochondria, since these pathways are the earliest acquisitions from a non–alpha-proteobacterial origin and have been widely conserved afterwards.

## Materials and Methods

### Genome sequence data.

A total of 144 publicly available, complete proteome sequences was retrieved from the European Bioinformatics Institute (EBI) Proteome database as of January 2005 (http://www.ebi.ac.uk/proteome); no genome was discarded from the analysis. Additional eukaryotic species were included, namely Plasmodium falciparum from PlasmoDB (http://plasmodb.org); Candida albicans from CandidaDB (http://genolist.pasteur.fr/CandidaDB); Takifugu rubripes from Fugu Genome Project (http://www.fugu-sg.org); *Danio rerio* from Sanger Sequencing Project (http://www.sanger.ac.uk/Projects/D_rerio/); Neurospora crassa from Center for Genome Research (http://www.broad.mit.edu/annotation/genome/neurospora/Home.html); Homo sapiens, *Mus musculus,* and *Rattus norvergicus* from the International Protein Index (IPI) set at EBI (http://www.ebi.ac.uk/IPI); and Anopheles gambiae from Ensembl (http://www.ensembl.org/Anopheles_gambiae/index.html). For the eukaryotic species, the organellar genomes were included in the gene set per species.

### Mitochondrial proteomics data.

Sequences and annotation data for experimentally identified mitochondrial proteins in human and yeast were retrieved from the MitoProteome (http://www.mitoproteome.org) [25] and the *Saccharomyces* Genome Database (SGD; http://www.yeastgenome.org) [39] databases, as well as from the supplementary material in Sickmann et al. [24].

### Reconstruction of the proto-mitochondrial proteome.

The approach used here to reconstruct the proto-mitochondrial proteome is conceptually similar but performed in a larger scale and technically more advanced than what we used in our previous reconstruction. Besides doubling the number of genomes that were included in the phylogenetic analyses, we improved the phylogenomic pipeline according to recent advances in phylogenetic algorithms. Alignments were performed using the more reliable program MUSCLE [40] and, most importantly, a second filter based on ML trees as implemented in PhyML [41] was used.

For every protein encoded in each of the 11 alpha-proteobacterial genomes, Smith-Waterman comparisons [42] were used to retrieve from the complete proteomes a set of homologous proteins with a significant similarity (E < 0.01) and with a region of similarity covering more than 50% of the query sequence. The sets of homologs that included proteins from eukaryotic genomes were further analyzed. These sets were first limited to the most similar 250 sequences, and additional homologous proteins were added only if they belonged to a species not already present in the initial 250 sequences. Every set of homologous sequences was aligned using MUSCLE [40]. Protein families with a likely proto-mitochondrial origin were selected by a two-step procedure: First, Neighbor Joining (NJ) trees were generated using Kimura distances as implemented in ClustalW [43], using 100 samples to perform the bootstrap analyses. Resulting phylogenetic trees were scanned by an algorithm (see below) for partitions indicating a monophyly of eukaryotic and alpha-proteobacterial proteins, the resulting set of selected OGs is referred to as NJ-set. Secondly, all original alignments selected in the NJ-set were used to generate ML trees using PhyML version 2.1b1 [41], with a four-rate gamma-distribution model. The tree-scanning algorithm was used for a second time on these ML trees, and the resulting selected OGs confirmed the ML-set. Note that OGs included in the ML-set have an alpha-proteobacterial descent that is supported by both NJ and ML tree-reconstruction techniques. The ML-set is thus a subset of NJ-set and is expected to include the OGs with the strongest phylogenetic signal of an alpha-proteobacterial origin.

### Tree-scanning algorithms.

*Selection of OGs derived from the proto-mitochondrion*. Phylogenetic trees were scanned for partitions that contained eukaryotic and alpha-proteobacterial proteins. The algorithm generates all possible partitions of the tree by sequentially removing all of its edges. Every time an edge is removed, two partitions are generated, and only the one that contains the seed sequence, that is, the sequence on which the tree is based, is taken into account. A species code attached to each sequence in the partitions allows testing whether that particular partition meets a number of criteria. In our case, the scanning algorithm examined whether a partition contained only alpha-proteobacterial and eukaryotic proteins and no archaeal or non–alpha-proteobacterial bacterial proteins. However, we made an exception for gamma- and beta-proteobacterial proteins. They were allowed to be in the alpha-proteobacterial/eukaryotic partitions for reasons of coverage, because otherwise the R. americana set was recovered at very low levels. For instance, most of the ribosomal proteins in R. americana mitochondrial genome were not recovered when not allowing for the presence of gamma- and beta-proteobacteria in the partitions. To illustrate this, we have included two representative examples (see Supplementary Figure 1 and Supplementary Figure 2 at http://bioinfo.cipf.es/tgabaldon/mito_metabolism.html). The first figure on that page of our Web site (http://bioinfo.cipf.es/tgabaldon/mito_metabolism.html) shows an example in which the selected partition (dashed square selection) contains only alpha-proteobacterial and eukaryotic species. In the example shown in Supplementary Figure 2 on our Web site, four gamma-proteobacterial sequences that fall within an alpha-proteobacterial/eukaryotic cluster are included in the partition.

In case an alpha-proteobacterial/eukaryotic partition existed, proteins in that branch were regarded as orthologs. The group was further divided into separate OGs if the proteins from the alpha-proteobacteria formed different “sister” subpartitions with the eukaryotic ones. We filtered out possible cases of horizontal gene transfer not related to the endosymbiosis, e.g., because they show signs of having been transferred from the eukaryotes to the alpha-proteobacteria, or because the transfer shows signs of having occurred very recently: (1) If a single alpha-proteobacterial protein was found within a cluster of eukaryotic proteins, this was interpreted as a gene transfer from a eukaryote to the alpha-proteobacteria and the group was discarded. (2) We also discarded cases in which only one genus of both eukaryotes and alpha-proteobacteria was present, eliminating proteins such as the ADP/ATP translocases that are only shared between the parasitic *Rickettsia* and Encephalitozoon cuniculi. Finally, new proteins were added to the selected OGs by the OG extension algorithm (see below).

As expected, varying the parameters, such as setting more stringent cutoffs, did reduce the number of selected OGs. For instance, increasing the requirement for species coverage such that two genera from both alpha-proteobacteria and eukaryotes were present in the OG discarded 112 (13.3%) groups from the final ML-set. Using a lower e-value threshold (<10^−5^) eliminated 233 (27.7%) groups from the ML-set. Setting a cutoff regarding the ratio between alpha-proteobacterial and beta- and gamma-proteobacterial sequences in the OG eliminated 60 (7.1%) groups from the ML-set, when a ratio higher than 1:1 was required, and 121 (14.3%), when a 2:1 ratio or higher was required.

Combining the more stringent cutoffs mentioned above reduces the number of OGs that can be traced back to an alpha-proteobacterial ancestor to only 203. However, this does not change qualitatively the results in terms of the functional classes that are preferentially retained in the mitochondrion, because after applying these cutoffs to modern mitochondria, most of what is left from the proto-mitochondrion, like in the original ML-set, belongs to energy metabolism, translation, or related functional classes. Of the 53 protein families from this highly stringent set that are mitochondrial in yeast and/or in human and for which there is a functional annotation, 26 (49.1%) and 14 (26.4%) belong to energy metabolism (C) and translation and post-translational modification (J and O) functional classes, respectively. The rest belong to other metabolic classes such as fatty acid metabolism (7.5%), amino acid transport and metabolism (7.5%), or coenzyme metabolism (3.8%), whereas functional classes involved in replication, transcription, and cell division are not represented.

*OG extension algorithm.* We noticed that, in many cases, true members of the selected OGs were not included in the selected tree partition. This was often caused by a single sequence from an unrelated bacterial species (not belonging to the alpha, beta, or gamma subdivisions of proteobacteria), which branched within the OG, potentially from a horizontal gene transfer to that species. This caused some true members from the selected OG to fall out of the selected partition. In order to minimize this effect, we extended the selected tree partitions to include close eukaryotic sequences in the OG if the number of sequences from unrelated species in the extended partition represented less than one-fifth of the total number of sequences from alpha-proteobacterial species. Note that this extension algorithm, which was applied to both NJ- and ML-sets, does not affect in any manner the number of selected OGs nor the reconstructed proto-mitochondrial metabolism. Hence, its effect is limited to the species coverage of previously selected OGs. Selected OGs, trees, and the list of genomes used in the analysis are available as supplementary material at http://bioinfo.cipf.es/tgabaldon/mito_metabolism.html.

*Metabolic reconstructions.* Annotated biochemical and cellular functions of the mitochondrial proteins or proto-mitochondrial–derived OGs were mapped onto metabolic KEGG maps [17] and assigned to a functional class as defined by the COG database [44]. In the case of the proto-mitochondrial metabolism, only pathways that have several consecutive steps represented in the ML-set were included. Additional adjacent reactions were added if they were present in the NJ-set. In the case of the human proteome, we excluded enzymes from the glycolytic pathway that is present in this set, as they likely result from contamination [26]. More detailed information of the pathways that are present in each proteome can be accessed through the supplementary material Web site accompanying this paper: http://bioinfo.cipf.es/tgabaldon/mito_metabolism.html.

### GO term analyses.

The program Fatigo+ from the Babelomics suite [45] was used to find specific and significantly overrepresented terms in the different proteomics fractions from the yeast and human mitochondrion. Each proteome was divided into four fractions (Figure 4): (1) proto-mitochondrial–derived, specific for that species; (2) proto-mitochondrial–derived, common to both species; (3) not derived from the proto-mitochondrion, common to both species; and (4) not derived from the proto-mitochondrion, species specific. The different sets were compared to the rest of the given mitochondrial proteome (e.g., a versus b + c + d). The fractions “human non-mitochondrial” and “yeast non-mitochondrial” correspond to the proto-mitochondrial–derived sets that are not mitochondrial in their respective species; these sets were compared with the total set of mitochondrial proteins to find overrepresented GO terms in the fraction of proteins that have been relocalized outside mitochondria. In Figure 4 are represented those terms that are (1) significantly overrepresented according to the adjusted *p*-value using the False Discovery Rate (FDR) procedure (in bold) and (2) specific for a given set and appear in three or more proteins.

## Abbreviations

ML: maximum likelihood
NJ: neighbor joining
OG: orthologous group
